# In this study: Adapting to the new normal in COVID-19 pandemic; a global survey & literature review

**DOI:** 10.1192/j.eurpsy.2021.280

**Published:** 2021-08-13

**Authors:** F. Arain, A. Tohid, A. Arain, D. Adam, F. Khan, A. Talpur, A. Arain, W. Azeem

**Affiliations:** 1 Psychiatry, BronxCare Health System Mount Sinai, NY, United States of America; 2 Psychiatry, University of Southern California, Los Angeles, United States of America; 3 Psychology, City College of New York, New York, United States of America; 4 Psychiatry, ICNA Relief Medical Clinic, Dallas, United States of America; 5 Family Medicine, Brooklyn Hospital, New York, United States of America; 6 Psychiatry, University of Louisville, Kentucky, United States of America; 7 Internal Medicine, Ziauddin University Hospital, Karachi, Pakistan; 8 Psychiatry, Sidra Medicine, Weill Cornell Medical College, Doha, Qatar, Doha, Qatar

**Keywords:** Covid-19, behavioral changes, life style modifications

## Abstract

**Introduction:**

Globally, governments have enforced protective measures of social distancing to prevent COVID-19 spread. The lifestyles of public have essentially transformed due to these actions. This study evaluates the effects of COVID-19 on connections and behavior/life adaptations.

**Objectives:**

Changes in life style and behavior in COVID-19-Pandemic

**Methods:**

We conducted a global cross-sectional study via survey on phone apps and social media platforms in population aged ≥ 16, including questions regarding demographic data and lifestyle changes. We also searched databases APA PsycNet, PubMed, PsycINFO, and Medline; reviewed 40 articles and included 3 in this review, a cross-sectional online survey^1^, a planned questionnaire^2^, and a study on 600 adolescents, age 10-19 in Palestine^3^

**Results:**

Our survey data showed total of 1002 responses, 31.7% decreased sleep, 42.1% increased appetite, 70.6% bulk-buying, and 50.2% weight gain. 43.1% less socialization than before, 78.7% increased screen time, 53.5% excessive hand washing/wiping surfaces, 45% reported social distancing facilitated in overpowering the fear of contracting infection, 29.4% negative impact on relationships, 80.7% noticed changes in behavior including shaking hands/hugging/speaking with a mask on, 49.5% adopted new hobbies, 34.9% showed increase in meditation. The literature review revealed that since COVID-19, there is an increase in screen time, weight, appetite, sleep, and a decrease in physical activity^1-3,^ and greater adherence to the Mediterranean diet in younger population^2^

**Conclusions:**

COVID-19 induced quarantine has caused increased screen time, appetite, weight gain, adoption of new hobbies, bulk-buying, hand washing, meditation, reduced sleep, and negatively impacted interaction/relationships. COVID-19 pandemic is ongoing and our data needs further assessment in more population studies.

**Disclosure:**

No significant relationships.

